# Photothermal‐Triggered Sulfur Oxide Gas Therapy Augments Type I Photodynamic Therapy for Potentiating Cancer Stem Cell Ablation and Inhibiting Radioresistant Tumor Recurrence

**DOI:** 10.1002/advs.202304042

**Published:** 2023-08-09

**Authors:** Tianfu Zhang, You Pan, Meng Suo, Meng Lyu, Jacky Wing Yip Lam, Zhaokui Jin, Shipeng Ning, Ben Zhong Tang

**Affiliations:** ^1^ School of Biomedical Engineering Guangzhou Medical University Guangzhou 510182 China; ^2^ Department of Chemistry the Hong Kong Branch of Chinese National Engineering Research Center for Tissue Restoration and Reconstruction and Guangdong‐Hong Kong‐Macro Joint Laboratory of Optoelectronic and Magnetic Functional Materials The Hong Kong University of Science and Technology Clear Water Bay Kowloon Hong Kong 999077 China; ^3^ Guangxi Medical University Cancer Hospital Nanning 530000 China; ^4^ Department of Gastrointestinal Surgery & Department of Geriatrics Shenzhen People's Hospital (The Second Clinical Medical College, Jinan University, The First Affiliated Hospital, Southern University of Science and Technology) Shenzhen Guangdong 518020 China; ^5^ School of Science and Engineering Shenzhen Institute of Aggregate Science and Technology The Chinese University of Hong Kong Shenzhen Guangdong 518172 China

**Keywords:** aggregation‐induced emission, cancer recurrence, cancer therapy, gas therapy, Type I photodynamic therapy

## Abstract

Despite advances in cancer therapy, the existence of self‐renewing cancer stem cells (CSC) can lead to tumor recurrence and radiation resistance, resulting in treatment failure and high mortality in patients. To address this issue, a near‐infrared (NIR) laser‐induced synergistic therapeutic platform has been developed by incorporating aggregation‐induced emission (AIE)‐active phototheranostic agents and sulfur dioxide (SO_2_) prodrug into a biocompatible hydrogel, namely TBH, to suppress malignant CSC growth. Outstanding hydroxyl radical (·OH) generation and photothermal effect of the AIE phototheranostic agent actualizes Type I photodynamic therapy (PDT) and photothermal therapy through 660 nm NIR laser irradiation. Meanwhile, a large amount of SO_2_ is released from the SO_2_ prodrug in thermo‐sensitive TBH gel, which depletes upregulated glutathione in CSC and consequentially promotes ·OH generation for PDT enhancement. Thus, the resulting TBH hydrogel can diminish CSC under 660 nm laser irradiation and finally restrain tumor recurrence after radiotherapy (RT). In comparison, the tumor in the mice that were only treated with RT relapsed rapidly. These findings reveal a double‐boosting ·OH generation protocol, and the synergistic combination of AIE‐mediated PDT and gas therapy provides a novel strategy for inhibiting CSC growth and cancer recurrence after RT, which presents great potential for clinical treatment.

## Graphical abstract

A near‐infrared‐induced therapeutic platform is developed by combining aggregation‐induced emission (AIE) material‐mediated phototherapy and sulfur dioxide (SO_2_) gas therapy. By the photothermal effect of AIE‐active TDCAc, SO_2_ is released and depletes upregulated glutathione in cancer stem cells (CSC), thus promoting hydroxyl radical (·OH) generation for enhancing photodynamic therapy. A double‐boosting ·OH generation protocol is revealed for inhibiting CSC growth and cancer recurrence after radiotherapy.

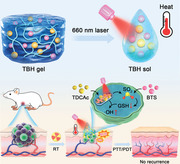

## 1 Introduction

Cancers, especially malignant and cancerous ones, are the leading causes of death worldwide. Radiotherapy (RT) has played a critical role in cancer treatment that directly causes DNA damage by ionizing radiation or oxidative damage by reactive oxygen species (ROS).^[^ ^1^, ^2^ ^]^ However, resistance to RT occurs frequently in the clinic, which can lead to treatment failure or cancer recurrence.^[^ ^3^, ^4^ ^]^ Although the underlying mechanism remains uncertain, the existence of cancer stem cells (CSC), considered as the “seeds” of cancer with self‐renewal, differentiation capacity and unlimited proliferative capacity, is widely believed to be a major cause of tumor relapse and metastasis after RT.^[^ ^5^, ^6^ ^]^ Different from non‐stem cancer cells, increasing experimental and clinical studies have provided evidence that CSC can survive high doses of radiation in various pathways, such as adjusting the cell cycle, repairing DNA damage, and modifying their division patterns.^[^ ^7^, ^8^ ^]^ The innate stemness endows them with radioresistance and eventually evades the cytotoxic effects of radiation. Therefore, developing a new treatment method that could overcome CSC resistance and solve the severe clinical challenges of recurrence and metastasis post‐radiotherapy is highly desirable.

Phototherapies, including photodynamic therapy (PDT) and photothermal therapy (PTT), are emerging as cutting‐edge modalities for cancer treatment with the virtues of light‐controllable capacity, noninvasiveness, and high spatiotemporal resolution.^[^ ^9^, ^10^, ^11^ ^]^ Numerous efforts have been made to develop phototheranostic agents.^[^ ^12^, ^13^, ^14^, ^15^ ^]^ However, the efficiency of these therapies is still far from ideal. For example, the hypoxic nature of the tumor microenvironment limited the ROS generation of PDT, while the acquired heat shock effect in PTT is another barrier.^[^ ^16^, ^17^, ^18^ ^]^ What's worse, conventional organic materials often suffer from aggregation‐caused quenching (ACQ) effect due to their planar structures, which hamper their energy transformation in the aggregate state and further limit the ROS generation and photothermal conversion efficiency.^[^ ^19^, ^20^ ^]^ Fortunately, fluorescence materials with aggregation‐induced emission (AIE) characteristics were developed as ideal options for phototheranostics owing to their facile structural tuning, extraordinary ROS generation and photothermal conversion efficiency at the aggregate state, thus enabling in vivo cancer treatment.^[^ ^21^, ^22^, ^23^, ^24^ ^]^ Recently, some AIE phototheranostic agents were reported to show the Type I PDT modality.^[^ ^25^, ^26^, ^27^ ^]^ Being empowered with the capability of radical ROS production with low dependence on oxygen content, the Type I AIE photosensitizer has emerged as a powerful alternative to overcome the inherent hypoxia nature of solid tumors, which shows potential for eliminating CSC in tumors. However, the utilization of phototheranostic agents in CSC treatment was rarely reported.^[^ ^28^ ^]^ The reason is that the extremely high level of intracellular glutathione (GSH) in CSC acts as an essential intracellular antioxidant for scavenging ROS, which becomes a “stumbling block” for diminishing the phototherapeutic effect.^[^ ^29^, ^30^ ^]^ Therefore, maintaining sustained ROS output during the Type I PDT process becomes a critical problem in guaranteeing therapeutic efficacy for CSC.

Gas therapy based on gaseous molecules is a novel method for promoting other cancer treatments for synergistic therapy.^[^ ^31^ ^]^ Specifically, several types of gaseous molecules, such as hydrogen,^[^ ^32^, ^33^, ^34^, ^35^ ^]^ hydrogen sulfide^[^ ^36^, ^37^ ^]^ and carbon monoxide (CO)^[^ ^38^, ^39^ ^]^ have been shown to sensitize cancer cells and synergistically boost PDT and RT. Sulfur dioxide (SO_2_) has traditionally been regarded as an air pollutant.^[^ ^40^ ^]^ Although SO_2_ has a toxicology effect during inhalation, its severe oxidative stress results in GSH depletion, which can be applied to increase intracellular ROS and induce cancer cell apoptosis. Focusing on the oxidative properties of SO_2_, we anticipated that SO_2_ plays a vital role in combating CSC in cancer therapy.^[^ ^41^, ^42^, ^43^ ^]^ Unfortunately, most of the reported SO_2_ therapeutic systems so far mainly focus on the therapy of the simple subcutaneous tumor model and have shown little effect on cancer recurrence due to the existence of CSC.^[^ ^44^, ^45^, ^46^ ^]^ In fact, tumor recurrence frequently occurs even after complete tumor removal.

**Figure 1:**
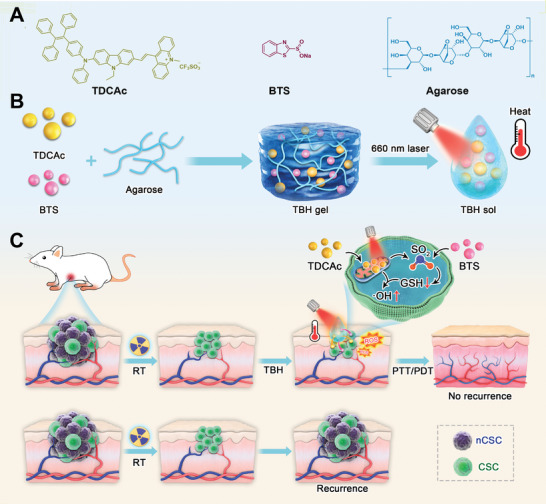
A) Chemical structures of components in TBH gel. B) Schematic illustration of the preparation of TBH gel with thermo‐responsive. C) Photothermal‐induced intracellular localized SO_2_ generation to enhance phototherapeutic action of TDCAc for killing CSC and inhibiting tumor recurrence after RT.

To address the problem, we herein reported the design of a thermo‐responsive SO_2_ generator to boost Type I AIE photosensitizer for effective ablating CSC and inhibiting tumor recurrence after RT. First, a multi‐modal AIE phototheranostic agent, TDCAc aggregates, and an SO_2_ donor, benzothiazole sulfinate (BTS),^[^ ^47^ ^]^ were co‐loaded into an injectable hydrogel to afford a multi‐functional system, namely TBH (**Figure** **1**A,B). TBH gel was rapidly heated up by the photothermal effect of TDCAc upon 660 nm laser irradiation, which dissolved and released the BTS and TDCAc aggregates in the tumor area. TDCAc was actively targeted to the mitochondria of both CSC and non‐stem cancer cells (nCSC) due to its cationic property and performed Type I PDT and PTT under continuous laser exposure. Afterward, the generation of intracellular SO_2_ from photothermal‐responsive BTS depleted GSH levels in CSC, which promoted the massive production of ·OH from PDT and caused CSC death. In both in vitro and in vivo experiments, the ablation of CSC was achieved, resulting in the inhibition of tumor recurrence in mice subjected to a combination treatment of TBH post‐RT. Conversely, mice treated solely with RT exhibited the growth of CSC and subsequent tumor recurrence. The combination of SO_2_ gas therapy and the multi‐model phototherapy based on the AIE molecule affords a groundbreaking approach for double‐boosting ·OH production, achieving a “1+1>2” synergistic anti‐tumor treatment (Figure 1C). Notably, the synergistic system represents a viable strategy for CSC treatment and preventing the recurrence of radioresistant tumors.

## 2 Results and Discussion

### 2.1 Fabrication and Characterization of TBH Hydrogel

**Figure 2:**
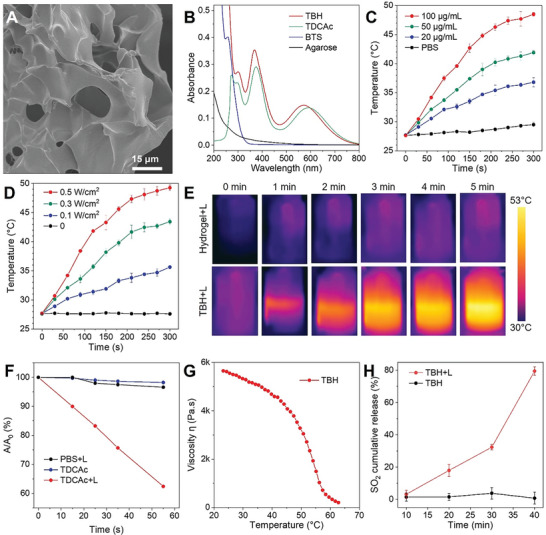
A) SEM image of TBH gel. B) Absorption spectra of pure agarose hydrogel, BTS, TDCAc and the TBH gel. C) Temperature elevation of TBH gel at different loading concentrations of TDCAc under 660 nm laser irradiation (0.5 W cm^−2^). D) Temperature elevation of TBH gel at 100 µg mL^−1^ of TDCAc under 660 nm laser irradiation with different power densities. E) Infrared thermal images of PBS solution and TBH gel upon exposure to 660 nm laser irradiation (0.5 W cm^−2^) at different times. F) The decomposition rate of methylene blue in the presence of PBS or TDCAc (10 µg mL^−1^) with or without laser irradiation (0.1 W cm^−2^). G) Rheological measurements of the viscosity of TBH gel at different temperatures. H) Fluorescence intensities of the DEACA probe after incubation with TBH gel with or without laser irradiation (0.5 W cm^−2^) for different periods of time. The concentration of TDCAc and BTS in the final prepared TBH gel is 100 µg mL^−1^ and 10 µg mL^−1^, respectively.

First, the AIE molecule TDCAc was synthesized facilely according to our previous report and fully characterized (Scheme S1, Supporting Information).^[^ ^48^ ^]^ Agarose hydrogel with high biocompatibility and the thermo‐sensitive property was used as a base to encapsulate TDCAc and BTS by a simple blending method to fabricate the synergistic therapeutic system. The resulting functional hydrogel named TBH showed a gel state at room temperature (Figure S1A, Supporting Information). The structure of the prepared TBH hydrogel was measured via scanning electron microscopy (SEM). The TBH gel was homogenously porous with a 3D pore size of 10–20 µm, which is suitable for drug loading and release (**Figure** **2**A). While element energy‐dispersive spectroscopy (EDS) analysis of the TBH gel shows prominent peaks of Na, S, and F elements, indicating the successful loading of BTS and TDCAc (Figure S2, Supporting Information). In addition, the transmission electron microscope (TEM) images displayed the spherical shape of nanoaggregates, which comes from TDCAc (Figure S3, Supporting Information). The absorption spectra of TBH gel confirmed the abundant loading of TDCAc aggregates, showing an absorption maximum at ≈575 nm (Figure 2B). The slightly blue‐shifted absorption of TBH was observed compared to that of TDCAc aggregates (590 nm), which could result from the weakened intramolecular charge transfer effect of TDCAc in the hydrophobic environment of the hydrogel. The strong absorption of TBH in the near‐infrared (NIR) region encouraged our subsequent exploration of photophysical properties under 660 nm NIR laser irradiation. As shown in Figure 2C and D, the temperature changes of TBH gel with different loaded TDCAc concentrations (0, 20,50, and 100 µg Ml^−1^) and laser power densities (0, 0.1, 0.3, and 0.5 W cm^−2^) were measured so that the drug loading condition of TBH could be optimized. The temperature of TBH gel was increased by over 20 °C under laser irradiation (Figure 2E) along with hydrogel dissolution (Figure S1B, S4, Supporting Information), which reveals an apparent photothermal conversion capability. Additionally, the ROS generation ability of TDCAc was testified by using 1,3‐Diphenylisobenzofuran as the singlet oxygen (^1^O_2_) indicator and methylene blue as the ·OH indicator. Figure 2F, Figure S5,S6 (Supporting Information) show that TDCAc produced a large amount of ·OH and ^1^O_2_ simultaneously, making it a Type I and Type II photosensitizer. To further verify the free‐radical ROS generation, 5,5‐dimethyl‐1‐pyrroline N‐oxide (DMPO) as a spin‐trap agent was used in electron spin resonance (ESR) measurements to assess the formation of free radicals (Figure S7, Supporting Information).^[^ ^49^, ^50^, ^51^ ^]^ Significant ESR signal could be observed in TBH gel and TDCAc upon irradiation, associated with the generation ability of ·OH. In contrast, the control group in the dark produced no signal. These results were in accordance with the previous work. The Rheology testing of TBH indicated the reduction of the storage modulus at an increasing temperature under laser irradiation, as illustrated by a decrease in viscosity (Figure 2G). This property makes this thermo‐sensitive hydrogel suitable for subsequent drug release. In general, the SO_2_ prodrug in this study, BTS, was reported to release in water slowly with pH dependence, which significantly hampers its in vivo efficiency due to the fast metabolism of the vascular system. Thus, TBH with photothermal capability was used here to accelerate BTS activation. The SO_2_ release was confirmed using a commercially available fluorescent probe, 7‐diethylaminocoumarin‐3‐aldehyde (DEACA), which can react with bisulfite anion to exhibit blue fluorescence at 483 nm. The enhancing fluorescence from DEACA indicates that the release of SO_2_ from the encapsulated BTS in TBH gel can be completed by thermal stimulation (Figure 2H).

### 2.2 In Vitro Therapeutic Studies of TBH upon NIR Light Irradiation

**Figure 3:**
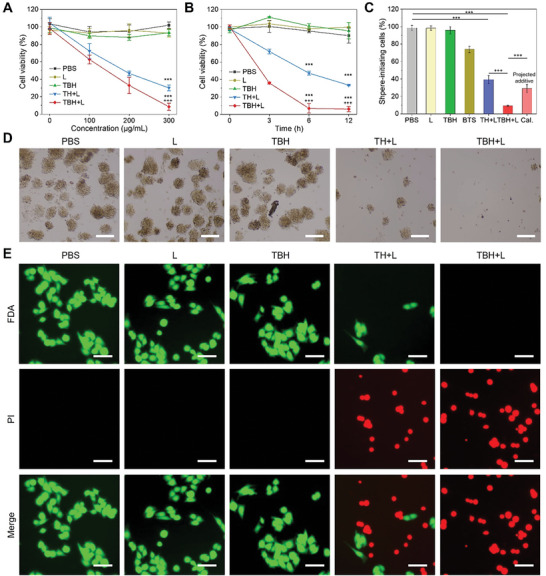
A) The cell viability of mixture 4T1 CSC and nCSC treated with PBS or two hydrogels containing different loading concentrations of TDCAc. B) The cell viability of mixture 4T1 CSC and nCSC treated with different formulations under different incubation times. C) The percentage of CSC tumor spheres after various treatments. The projected additive value is calculated by multiplying the surviving fraction of cells from the independent BTS treatment and the surviving fraction of the TH+L treatment. D) Sphere‐formation assays using 4T1 CSC cultured with various treatments. Scale bars: 100 µm. E) Live/dead cell staining of 4T1 cells after various treatments, live cells were stained with green fluorescent FDA and dead cells were stained with red fluorescent PI. Scale bar: 60 µm. The concentration of TDCAc and BTS in the final prepared TBH or TH gel is 100 µg mL^−1^ and 10 µg mL^−1^, respectively. The applied parameter of 660 nm laser irradiation is 0.5 W cm^−2^ for 5 min. Data are presented by mean ± SD, *n* = 3, **p< 0.01, ***p< 0.001 as compared with the controlled group, ^++^p< 0.01, ^+++^p< 0.001 as compared with the “TH+L” group.

The remarkable capabilities of TBH gel in ROS generation, photothermal conversion, and gas release highlight its potential for combined cancer therapy. The potential cytotoxicity and treatment feasibility of TBH was investigated by standard methyl thiazolyl tetrazolium (MTT) assay. TH hydrogel was constructed without BTS as a reference. Under laser irradiation, both TH and TBH hydrogels gradually softened due to the photothermal effect. The viability of 4T1 cells maintained high when incubated with the TBH without laser (L) irradiation, indicating its excellent biocompatibility. In contrast, the cell viability of the “TH+L” and “TBH+L” groups gradually decreased to 30% and 8% with 300 µg Ml^−1^ of TDCAc in the gels (**Figure** **3**A). In addition, the cell viability of TBH showed the most efficient cytotoxicity by killing ≈95% of cancer cells within only 6 h of incubation (Figure 3B). For deep research of tumor therapy, CSC should be taken into consideration. The existence of CSC can cause cancer relapse and radiation resistance through their ability to arrest in a relatively static phase, which is the main reason for treatment failure. After extracting and isolating the 4T1 CSC with high expression of CD133 under optimized culture conditions (Figure S8, Supporting Information), we further evaluated CSC proliferation using a stem cell spheroidization assay to understand the therapeutic effect deeply. As shown in Figure 3C,D, the spheroid formation rate of the TH+L group remained at a high level of ≈40% despite single phototherapy. In comparison, most of the spheroid was removed in the “TBH+L” group, with a spheroid formation rate of <5%. Figure 3C shows that the combined therapy in TBH gel exhibited significantly higher cytotoxicity than the two independent therapies. Gas therapy using BTS alone caused ≈26% of cell death (74% cell viability), while the TH+L group using AIE photo‐induced therapy alone caused ≈61% of cell death (39% cell viability). However, by combining the two into a system upon light irradiation, TBH caused about 91% of cell death (9.24% cell viability), showing a stronger cancer cell‐killing effect than the projected additive model developed by Hahn.^[^ ^52^, ^53^ ^]^ This suggests that SO_2_ gas therapy and AIE‐mediated phototherapy in TBH gel act synergistically under certain conditions. To demonstrate the cytotoxicity intuitively, live/dead cell double‐staining approach was performed by using fluorescein diacetate (FDA) and propidium iodide (PI), which can distinguish live and dead cancer cells from green and red fluorescence. As expected, a strong red fluorescent signal was observed in the “TBH+L” group. In contrast, 4T1 cells presented healthy state (green) in the other used laser and pure TBH hydrogel control group (Figure 3E). Collectively, TBH has a NIR laser‐controllable cytotoxic effect on nCSC and CSC.

## 3 Mechanistic Studies of the Therapeutic Action in nCSC and CSC

**Figure 4:**
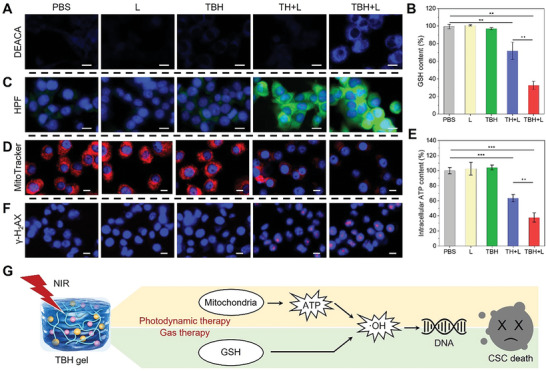
A) Intracellular SO_2_ generation in 4T1 cells in different formulations with or without laser irradiation. DCACA was used as an SO_2_ indicator. Scale bar: 15 µm. B) Intracellular GSH level in mixture 4T1 CSC and nCSC treated with different formulations. C) Intracellular ·OH in 4T1 cells after various treatments with or without laser irradiation. HPF was used as ·OH indicator. Scale bar: 15 µm. D) Mitochondria activity in 4T1 cells after various treatments with or without laser irradiation. MitoTraker Red was used as mitochondria targeting dyes. Scale bar: 10 µm. E) Intracellular ATP level in mixture 4T1 CSC and nCSC treated with different formulations. F) Detection of DNA damage by γ‐H_2_AX assay in 4T1 cells after various treatments. Scale bar: 10 µm. G) Proposed mechanism for combined PDT/gas therapy based on the TBL gel. The concentration of TDCAc and BTS in the final prepared TBH or TH gel is 100 µg mL^−1^ and 10 µg mL^−1^, respectively. Laser parameter: 660 nm, 0.5 W cm^−2^, 5 min. Data are presented by mean ± SD, *n* = 3, **p< 0.01, ***p< 0.001 as compared with the controlled group, ^++^p< 0.01, ^+++^p< 0.001 as compared with the “TH+L” group.

Cancer cells, especially for CSC, are highly adaptive to external oxidative stress from PDT and survive by upregulating GSH levels. The appropriate amount of SO_2_ was reported to directly annihilate GSH, thereby inhibiting the self‐protect process of CSC and promoting the ROS‐based PDT. To decipher the rationale behind CSC killing effect of TBH, the in vitro photoinduced release of SO_2_ from TBH was monitored first by using DEACA a probe (**Figure** **4**A). After irradiating with a NIR laser, an enhanced blue fluorescence signal was obtained in the cells treated with TBH gel. In contrast, nearly no fluorescence was observed in the cells from the other four groups, including the “TH+L” group, demonstrating that SO_2_ was controllably produced from TBS‐contained TBH by applying laser stimulus. Meanwhile, the intracellular GSH level was assessed and shown in Figure 4B. About 30% of GSH was consumed in the “TH+L” group due to the cancer cell death by the phototherapeutic effect of TDCAc. By loading the BTS in TBH and irradiating with an NIR laser, >60% intracellular GSH was reduced compared to the control, confirming the inhibition of GSH by SO_2_ generation. Afterward, the PDT effect by the TBH can induce the generation of large amounts of ROS in cells. By using hydroxyphenyl fluorescein (HPF) as an ·OH indicator, cells treated with TH and TBH gel under laser irradiation displayed prominent green fluorescence, while no apparent fluorescence could be seen in the other groups (Figure 4C; Figure S9, Supporting Information). This indicated increasing intracellular ROS accumulation by the PDT effect of TDCAc‐contained hydrogel. In addition, owing to the mitochondria‐targeting capability of TDCAc, laser‐triggered TBH severely impaired the mitochondria of 4T1 cells (Figure 4D; Figure S10, Supporting Information) and thus led to the irreducible inhibition in cellular energy level and reduced intracellular ATP (Figure 4E). As a result, such a remarkable effect of TBH on cancer cells caused DNA damage and CSC death (Figure 4F). Therefore, the excellent tumoricidal efficacy of TBH could be attributed to the synergistic effect of AIEgen‐mediated phototherapy and SO_2_ gas therapy (Figure 4G). Together with the excellent therapeutic performance on nCSC and CSC upon laser irradiation, TBH gel shows great potential for solving the radioresistance issue of tumors during RT.

### 3.1 In Vivo Synergistic AIEgen‐Mediated PDT and Gas Therapy for Killing CSC and Inhibiting Post‐RT Cancer Recurrence

**Figure 5:**
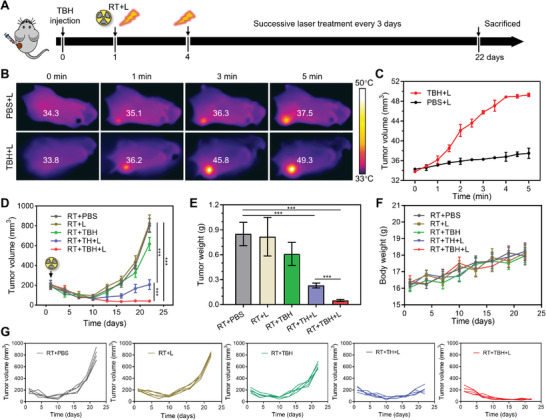
A) Schematic operation process of antitumor treatment by radiation and laser irradiation. B) Infrared thermal images of 4T1 tumor‐bearing mice under 660 nm laser irradiation (0.5 W cm^−2^) for different times after injection of TBH gel. C) The temperature change at the tumor area of the mice at different times of 660 nm laser irradiation (0.5 W cm^−2^). D) Tumor volume growth curves of mice at different times after treatments. E) The tumor weight measurement of mice on day 22 after treated with different formulations. F) Body weight measurements of mice in different groups. G) Tumor growth curve of each mouse in different groups as illustrated in (D). Data are presented by mean ± SD, *n* = 5, ***p< 0.001.

Although numerous AIE phototheranostic agents were developed for efficient multi‐modality cancer therapy, almost all the applied tumor models were simply cultured by a “bag” of homogeneous malignant cells, which could not represent intratumoral heterogeneity, such as the development of CSC. In light of the outstanding in vitro phototherapeutic performance of TBH, in vivo application feasibility of TBH for CSC and its potential to prevent tumor recurrence after RT were encouraged to be estimated. A subcutaneous 4T1 tumor‐bearing BALB/c mice model was established containing CSC and nCSC. The mice were randomly divided into five groups, which were named “RT+PBS”, “RT+L”, “RT+TBH”, “RT+TH+L”, and “RT+TBH+L”. When the tumor volumes grew to ≈200 mm^3^, all the mice were exposed to X‐ray irradiation (8 Gy). After the radiation exposure, PBS, TH, and TBH were given by intratumoral injection. The tumors were then subjected to phototherapy by 660 nm laser irradiation for 5 min every three days. Mice that received X‐ray radiation without laser were set as the control “RT+PBS” group (**Figure** **5**A). First, temperature elevation was monitored using infrared imaging with a thermal camera under 660 nm laser irradiation. As displayed in Figure 5B,C, the temperature of the tumor area treated with TBH elevated rapidly from 33.8 to 49.3 °C during 5 min of laser irradiation. While the PBS group showed negligible temperature change by only a 3.2 °C increase under the same condition. Proven to have in vivo photothermal effect, TBH could release TDCAc and SO_2_ to tumor areas after the local dissolution for in situ therapy. Figure 5D illustrated that the tumors were inhibited after RT treatment in the first few days. However, tumor volumes in the “RT+PBS”, “RT+L”, and “RT+TBH” groups had recurrent to the original level for ≈15 days after treatment (Figure 5E), with a dramatic increase in tumor weight and high expression of CD133 (Figure S11, Supporting Information) in ≈30 days post‐RT. Although tumor recurrence was limited by single phototherapy in the “RT+TH+L” group, the tumor still relapsed to ≈200 mm^3^ due to incomplete removal of self‐renewal CSC. In contrast, no tumor recurrence was observed in the “RT+TBH+L” group and the tumors were eliminated. The synergistic tumor inhibition effect of TBH is much stronger than the projected values of phototherapy and gas therapy (Figure S12, Supporting Information), indicating that combined TDCAc‐induced phototherapy and SO_2_ gas therapy with synergistic effect can kill CSC efficiently and remedy the defect of RT (Figure 5G; Figure S13, Supporting Information). Additionally, the body weights of all the mice in every group had no evident variation (Figure 5F).

### 3.2 In Vivo Biosafety of TBH

**Figure 6:**
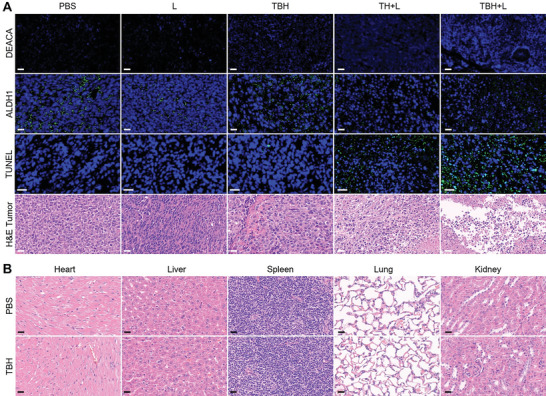
A) DEACA, ALDH1, TUNEL, and H&E of tumor slices collected after the different treatments. Scale bars: 30 µm. B) Histological analysis of H&E‐stained slices of the main organs of mice after injecting PBS and TBH gel. Scale bars: 15 µm.

To fully comprehend the therapeutic mechanism, the mice treated with the above formulations were sacrificed after treatment and SO_2_ production inside the tumor areas was determined by DEACA (first row of **Figure** **6**A). Different from all the other groups, a strong blue fluorescence signal from the indicator was observed in the “RT+TBH+L” group, confirming the increased SO_2_ release after activating the BTS prodrug in the TBH gel upon laser irradiation. Next, aldehyde dehydrogenase 1 (ALDH1) staining was used to evaluate the CSC viability. ALDH1 is a group of enzymes that is important for the maintenance and differentiation of CSC. It is reported that ALDH expresses during therapy and promotes radiation resistance and survival mechanisms in CSC. As shown in the third row of Figure 6A, the diminished expression levels of ALDH1 in the “RT+TBH+L” group indicate a significant therapeutic effect on CSC. Furthermore, The TUNEL assay and H&E staining results of the tumor slice demonstrated that the treatment of RT and TBH caused severe damage to cancer cells. In addition, the H&E staining results showed high hemocompatibility and no noticeable pathological morphology changes in the main organs (heart, liver, spleen, lung, and kidney), demonstrating the biological safety of TBH gel (Figure 6B; Figure S14, Supporting Information). Overall, these findings testified the biocompatibility and the high efficacy of synergistic phototherapy and SO_2_ gas therapy by TBH, which is promising for solving cancer recurrence issues after RT and other traditional treatments in the clinic.

## 4 Conclusion

In summary, we have successfully developed an intelligent NIR‐induced synergistic therapeutic platform named TBH by introducing AIE‐active phototheranostic agents and SO_2_ prodrug into a biocompatible hydrogel to overcome malignant CSC growth. Outstanding ·OH generation and photothermal effect of the AIE phototheranostic agent actualized Type I PDT and PTT for multi‐model cancer treatment. Thermo‐responsive BTS prodrug released an appropriate amount of SO_2_ by photothermal effect, which inhibited the self‐protection process of CSC by depleting intracellular upregulated GSH and consequentially promoted ·OH level of PDT. The resulting NIR‐induced TBH hydrogel could diminish CSC and nCSC simultaneously under 660 nm laser irradiation and ultimately inhibited the recurrence of the radioresistant tumor. In comparison, the tumor in the mice that were only treated with RT relapsed rapidly. Thus, double‐boosting ROS generation was accomplished by the synergistic protocol of gas therapy and AIE phototherapy, inhibiting CSC growth and cancer recurrence after RT. This combined synergistic therapy inspires further exploration of novel AIE‐active theranostic systems for potential clinical translation.

## Conflict of Interest

The authors declare no conflict of interest.

## Supporting information

Supporting Information

## Data Availability

The data that support the findings of this study are available from the corresponding author upon reasonable request.
