# Supplementary material for: Photothermal‐Triggered Sulfur Oxide Gas Therapy Augments Type I Photodynamic Therapy for Potentiating Cancer Stem Cell Ablation and Inhibiting Radioresistant Tumor Recurrence
Source: Adv Sci (Weinh). 2023 Aug 9;10(29):2304042. doi: 10.1002/advs.202304042 (PMC10582409; doi:10.1002/advs.202304042)
Supplement: Supplementary file 1 — Supporting Information [file ADVS-10-2304042-s001.pdf]

ADVANCED SCIENCE
Open Access

Supporting Information

for *Adv. Sci.*, DOI 10.1002/advs.202304042

Photothermal-Triggered Sulfur Oxide Gas Therapy Augments Type I Photodynamic Therapy for Potentiating Cancer Stem Cell Ablation and Inhibiting Radioresistant Tumor Recurrence

Tianfu Zhang, You Pan, Meng Suo, Meng Lyu, Jacky Wing Yip Lam, Zhaokui Jin*, Shipeng Ning* and Ben Zhong Tang**

Supporting Information

Photothermal-Triggered Sulfur Oxide Gas Therapy Augments Type I Photodynamic Therapy for Potentiating Cancer Stem Cell Ablation and Inhibiting Radioresistant Tumor Recurrence

Tianfu Zhang[#], You Pan[#], Meng Suo[#], Meng Lyu, Jacky Wing Yip Lam, Zhaokui Jin,* Shipeng Ning,* Ben Zhong Tang**

Materials and reagents

9-Methylacridine, 2,6-ditert-butyl-pyridine and trifluoromethanesulfonic acid were purchased from Sigma-Aldrich or TCI. Benzothiazole Sulfinate (BTS) was purchased from Henan Weitixi Chemical Technology Co. LTD. 3'-(4-Hydroxyphenyl) fluorescein (HPF) was purchased from Sigma-Aldrich (China). MTT Cell Proliferation Assay Kit was purchased from Yeasen Biotech Co., Ltd (China). All solvents and reagents used were purchased from commercial suppliers without further purification. Other reagents were purchased from Sinopharm Chemical Reagent (China) and Aladdin-Reagent (China). The aldehyde intermediate was synthesized according to our reported method.[1]

Synthesis of TDCAc

A solution of the aldehyde intermediate (0.10 g, 0.18 mmol) and 9-Methylacridine (60 mg, 0.34 mmol) were reflux in dry AcOH (1.5 mL) catalyzed by AcO_2 (1.0 mL) for 48 h under N_2. After cooling to room temperature, the reaction mixture was neutralized by sodium carbonate and extracted with DCM. The organic layer was dried over sodium sulfate and the solvent was removed by evaporation. The residue was purified by column chromatography eluting with ethyl acetate/hexane (10:1, v/v) and the final product was directly used in the next step. Subsequently, all the product was added in superdry DCM (3 mL) under N_2 with 4 equiv. of 2,6-ditert-butyl-pyridine and 5 equiv. of $MeCF_3SO_3$. After stirring at room temperature for 3 h, the solvent was evaporated under reduced pressure. The residue was run by a flash column eluted with DCM/MeOH (10:1, v/v) to give dark solid TDCAc (62 mg, 42%). ^{1}H NMR (400 MHz, d_6-DMSO, δ): 8.95 (d, J = 8.6 Hz, 1H), 8.81 (s, 1H), 8.76 (d, J = 9.2 Hz, 2H), 8.46-8.42 (m, 2H), 8.28 (s, 1H), 8.19 (d, J = 8.0 Hz, 1H), 8.12 (d, J = 8.4 Hz, 1H), 8.02 (t, J = 7.7 Hz, 2H), 7.84 (d, J = 8.1 Hz, 1H), 7.57 (d, J = 16.1 Hz, 1H), 7.35-6.83 (m, 27H), 4.80 (s, 3H), 4.38 (m,

2H), 1.30 (t, J = 7.2 Hz, 3H) HRMS (MALDI-TOF): m/z calcd. For $C_{62}H_{48}N_3^+$ [M-CF_3SO_3]$^+$: 834.3843; found: 834.3603.

Preparation of TBH hydrogel

The general protocol for the hydrogel preparation is as follows. The prepared TDCAc (0.5 mg) and BTS (50 μg) were mixed into 10 mL 2% agarose solution to form TBH, followed by freeze drying to obtain 5 mL TBH. Scanning electron microscopy (SEM) was conducted to characterize. SEM images were captured on a Hitachi FE-SEM S4800 instrument with an acceleration voltage of 3 kV. Rheology experiments were performed on HAAKE MARS60 (Thermo Fisher Scientific) according to our previous work[1].

SO_2 release

For the quantitative investigation of SO_2 release, 7-diethylaminocoumarin-3-aldehyde (DEACA) was selected as a fluorescent probe for bisulfite, which would change from non-fluorescent to blue fluorescent selectively owing to the nucleophilic addition reaction to the formyl functional group with bisulfite anion. The fluorescence spectrophotometer was utilized to measure the fluorescence intensity with the excitation of 390 nm.[2] 5 μM of DEACA solution was added into the 5 mL of TBH (containing 0.05 mg BTS and 0.5 mg TDCAc) solutions. Then a 660 nm laser (0.5W/cm^2) is introduced.

·OH detection

Methylene blue (MB) was used for ·OH detection. Firstly, 0.05 mL TDCAc (0.01 mg/mL) was added into 2 mL of MB, and the mixture was kept in the dark for 30 min. Subsequently, a 660 nm laser (0.1 W/cm^2) was employed as the excitation source. The absorption spectrum of the samples was measured at different times.

Photothermal effect

A 660 nm NIR laser (Changchun New Industries Tech.Co., Ltd., Changchun, China) with irradiation powers of 0.5 W/cm^2 was used to stimulate the different concentrations of TDCAc aggregates (20, 50 and 100 μg/mL) in aqueous solution. The photothermal curve and images during laser irradiation were recorded every 30 s using an infrared camera (Fotric 225). The photothermal effect of TDCAc aggregates (100 μg/mL) under the NIR laser irradiation with various power densities (0, 0.1, 0.3, and 0.5 W/cm^2) was evaluated in the same way.

Cell culture

4T1 cell lines were obtained from the Cell Bank of the Chinese Academy of Sciences and incubated in RPMI-1640 medium supplemented with 10% FBS in a humidified atmosphere.

The cancer stem cells (CSC) were sorted from 4T1 cells and cultured as guided by the previous report.[3] 4T1 cells suspension (10000 cells/ml) were seeded in ultra-low attachment surface 6-well plates (Corning, USA) with serum-free DMEM-F12 medium, containing B27 (1:50), epidermal growth factor, basic fibroblast growth factor (20 ng/mL), N_2 additives. Primary cultures of colonospheres were harvested after 7 days in culture and collected after centrifugation, dissociated with Trypsin–EDTA.

The Non-cancer stem cells (nCSC) were also sorted from 4T1 cells. Briefly, 4T1 cells were split in Serum Containing Medium (SCM). This is made up of DMEM/Ham's F12 (1:1) (Hyclone), 10 % Foetal Bovine Serum (FBS) (Biological Industries), 1 % penicillin/streptomycin (P/S) (100 U/ml) (Hyclone), and 1 % L-glutamine (LG) (Lonza). The adherent cells obtained from the SCM were harvested by trypsinization to obtain single cells and then expanded in SCM medium to obtain more cells. These cells were scored as non-cancer stem cells (nCSC).

Intracellular ·OH detection

4T1 cells (1×10^6 per plate) were incubated with 5 different groups: (1) PBS; (2) Laser (L) (660 nm, 0.5 W/cm^2, 5 min); and (3) TBH; (4) TH+L, and (5) TBH+L. The TDCAc concentration was 100 μg/mL. Then, the ·OH probe HPF was added (final concentration: 10 μM) into predetermined groups and incubated for 30 min. Subsequently, the cells were washed with PBS for five times and fixed with 4% formaldehyde for 10 min. The cells were observed by a fluorescence microscope.

Detection of intracellular SO_2

4T1 cells (1×10^6 per plate) were incubated with 5 different groups: (1) PBS; (2) L (660 nm, 0.5 W/cm^2, 5 min); and (3) TBH; (4) TH+L, and (5) TBH+L. The TDCAc concentration was 100 μg/mL. After 6 hours of incubation, the SO_2 probe DEACA was added (final concentration 10 μM) into predetermined groups and incubated for 30 min. Subsequently, the cells were washed with PBS for five times and fixed with 4% formaldehyde for 20 min. The cells were observed by a fluorescence microscope.

Detection of intracellular GSH and ATP

4T1 cells (1×10^6 per plate) were incubated with 5 different groups: (1) PBS; (2) L (660 nm, 0.5 W/cm^2, 5 min); and (3) TBH; (4) TH+L, and (5) TBH+L. The TDCAc concentration was 100 μg/mL. After 12 hours of incubation, the GSH and ATP content was measured respectively by employing a commercial colorimetric GSH assay kit and ATP assay kit from Beyotime Biotechnology, Shanghai, China. The assay was carried out according to the manufacturer's instructions.

***In vitro* anticancer effect of TBH**

The anticancer effect was measured by MTT assay. 4T1 cells (8×10^4 per plate) were incubated with 5 different groups: (1) PBS; (2) L (660 nm, 0.5 W/cm^2, 5 min); and (3) TBH; (4) TH+L, and (5) TBH+L with concentration was 0, 100, 200 and 300 μg/mL. At the end of the incubation, the cytotoxicity was calculated according to the MTT Kit instructions. Subsequently, we conducted a similar experiment by changing the irradiation time.

To further visualize the cell phototoxicity of each group, 4T1 cells were incubated for 24 h with 5 different groups: (1) PBS; (2) L (660nm, 0.5W/cm^2, 5min); (3) TBH; (4) TH+L; and (5) TBH+L with TDCAc concentration was 100 μg/mL. Next, cells were washed with PBS for 3 times, treated with FDA and PI according to the manufacturer's protocol, and performed the cell imaging under a fluorescent microscope (IX81, Olympus, Japan).

Mitochondrial integrity assay

4T1 cells were seeded into a 6-well plate and cultured overnight. 4T1 cells were pre-incubated incubated and treated with 5 different groups: ((1) PBS; (2) L (660nm, 0.5W/cm^2, 5min); and (3) TBH; (4) TH+L, and (5) TBH+L. The TDCAc concentration was 100μg/mL. After removing the residual nanomaterials, MitoTracker Red solution was added and incubated for 30 min. After removing the medium, the cells were washed thrice with PBS. Finally, the fluorescence images were recorded using CLSM.

Cytotoxicity in CSC-enriched 3D tumorsphere cells

Tumor spheres that were developed from 5000 CSC in 24-well ultralow attachment plates were treated with (1) PBS; (2) L (660 nm, 0.5 W/cm^2, 5 min); and (3) TBH; (4) TH+L, and (5) TBH+L. The TDCAc concentration was 200 μg/mL. The PDT was conducted after different treatments. After 5 days of incubation, take pictures of the tumorsphere and count the number.

Animal tumor models

Female BALB/c mice aged 4-5 weeks were purchased from Vital River Company (Beijing, China). 100 μL of 4T1 cell suspension (1×10^6 cells per mL) were subcutaneous injected into each mouse to construct the tumor models. The animal experiments were carried out according to the protocol approved by the Ministry of Health in the People's Republic of PR China and were approved by the Administrative Committee on Animal Research of the Shenzhen People's Hospital (AUP-210804-LM-001-0620).

***In vivo* infrared thermography**

To monitor the in vivo photothermal effect, TBH (TDCAc: 10 mg/kg) was intratumorally injected into the 4T1 tumor-bearing mice, and then the tumors suffered from 0.5 W/cm^2 irradiation for 10 min after 0.5 h of injection. PBS injection was used as a control group. Meanwhile, the temperature at the tumor site was monitored using an infrared camera (Fotric 225).

***In vivo* therapy.**

The 4T1 tumor model was used. When tumors reached 200mm^3, tumor-bearing mice were treated by X-ray irradiation (8 Gy). And then the mice were divided randomly into five different groups (n = 5): (1) PBS; (2) L (660 nm, 0.5 W/cm^2, 5 min); and (3) TBH; (4) TH+L, and (5) TBH+L. The TDCAc dose was 10 mg/kg. The irradiation was performed 0.5 h after injection. The treatment was conducted every three days. Mice's body weight and tumor volume were monitored every 3 days. After treatment, all the mice were sacrificed. The blood samples from these mice (≈1 mL) were collected for blood biochemistry analysis. Five main organs (heart, liver, spleen, lung and kidney) and tumors of all mice were harvested, washed with PBS, and fixed with paraformaldehyde for histology analysis. And the tumor tissues were weighed, and stained with DEACA, H&E and ALDH1 and finally examined by using a confocal laser scanning microscope (CLSM; IX81, Olympus, Japan).

Preliminary *in vivo* toxicity assessment

The female BALB/c mice (18–22 g) were intraperitoneally injected with PBS or TSH (10 mg/kg of TDCAc every 3 days. After 22 days from the first injection, the mice were sacrificed, and the heart, liver, spleen, lung, and kidney were harvested. The obtained tissues were fixed in formalin for the next paraffin sectioning and hematoxylin and eosin (H&E) assays to evaluate the histopathologic toxicity of the typical tissues.

Statistical analysis

Experimental data were analyzed by using one-way ANOVA followed by the post-Tukey comparison tests with GraphPad Prism 8.0 software. $P < 0.05$ indicates a statistical difference. $^{**}P < 0.005$, $^{***}P < 0.001$.

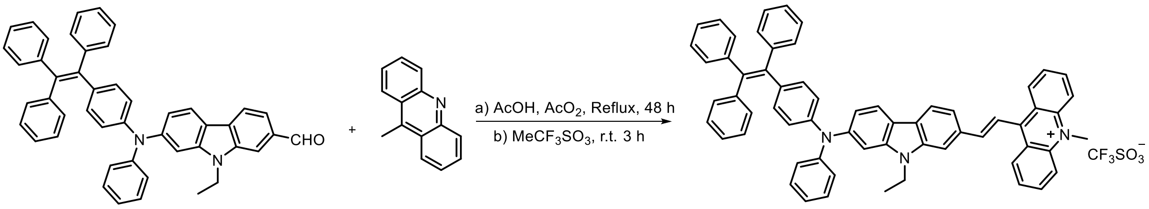

Scheme S1. Synthetic route of TDCAc.

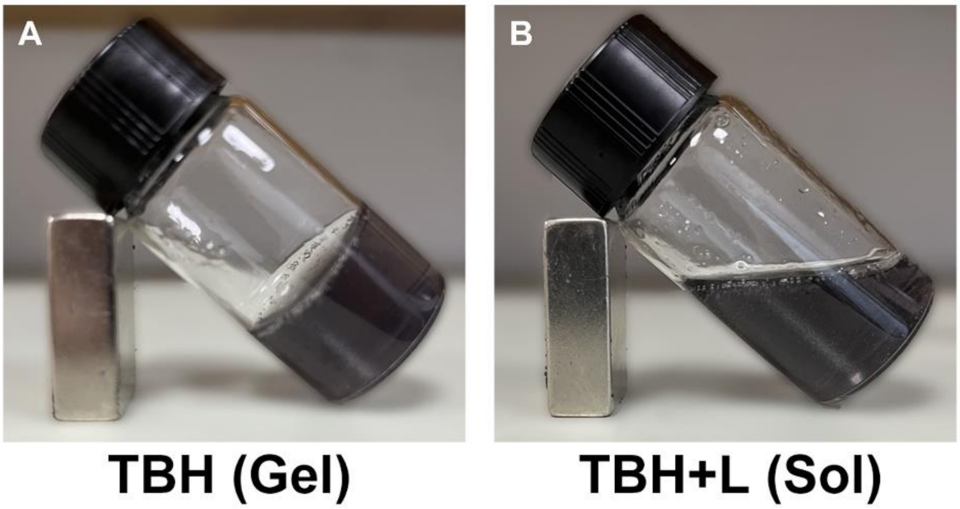

Figure S1. Photograph of TBH hydrogel (A) at the gelation state or (B) solution state after irradiated by 660 nm NIR laser.

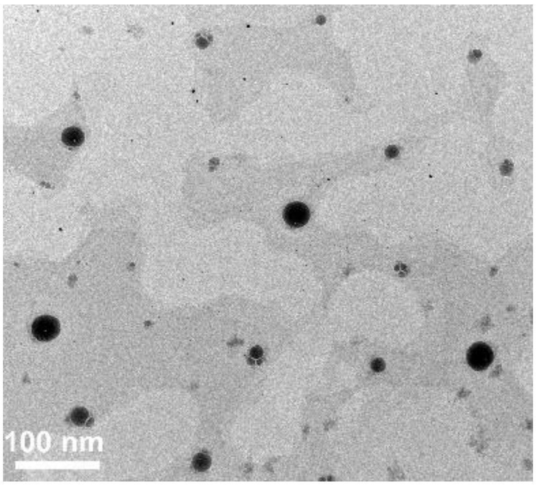

Figure S2. TEM images of TBH gel.

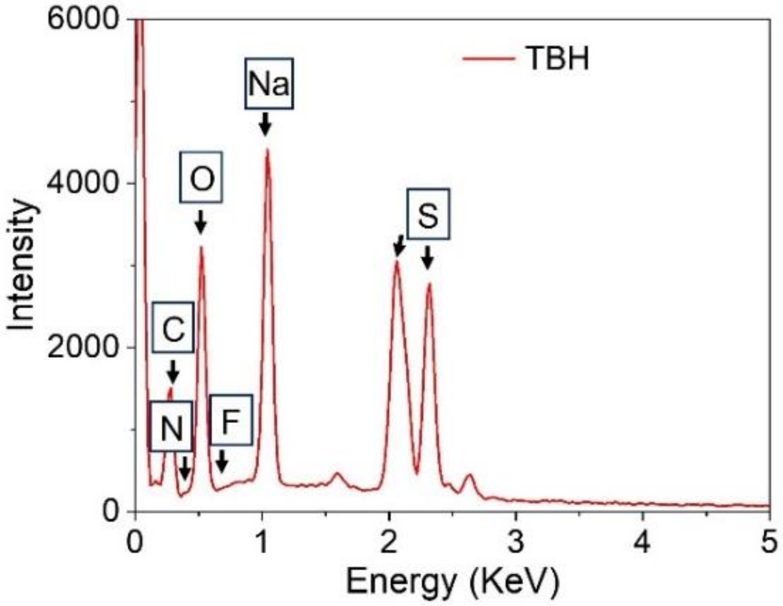

Figure S3. EDS spectrum of TBH gel.

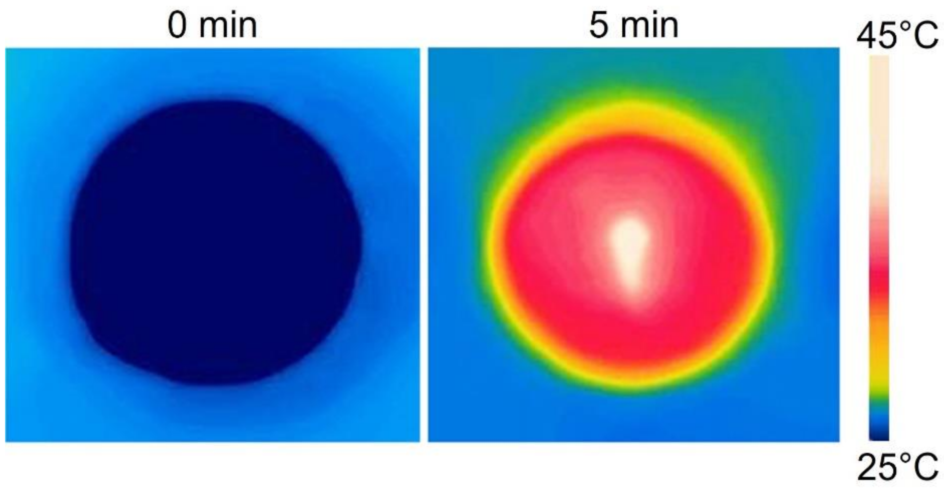

Figure S4. Infrared thermographic images of TBH hydrogel before and after laser irradiation (660 nm, 0.5 W/cm) for 5 min.

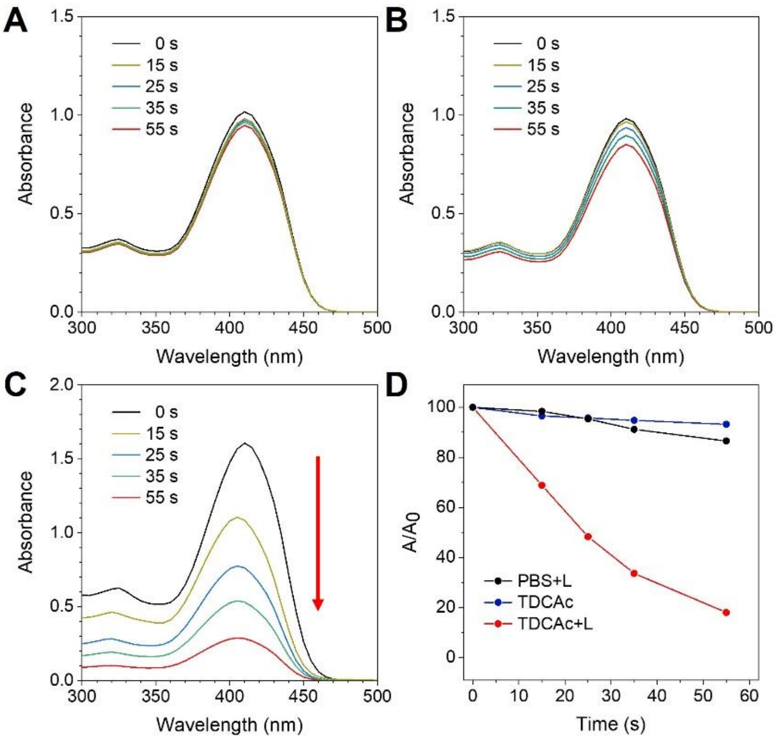

Figure S5. Absorption spectrum of 1,3-Diphenylisobenzofuran in the (A) PBS under laser irradiation and TDCAc solution in the (B) absence or (C) presence of irradiation for different times. (D) The decomposition rate of 1,3-Diphenylisobenzofuran by ·OH generation of these conditions.

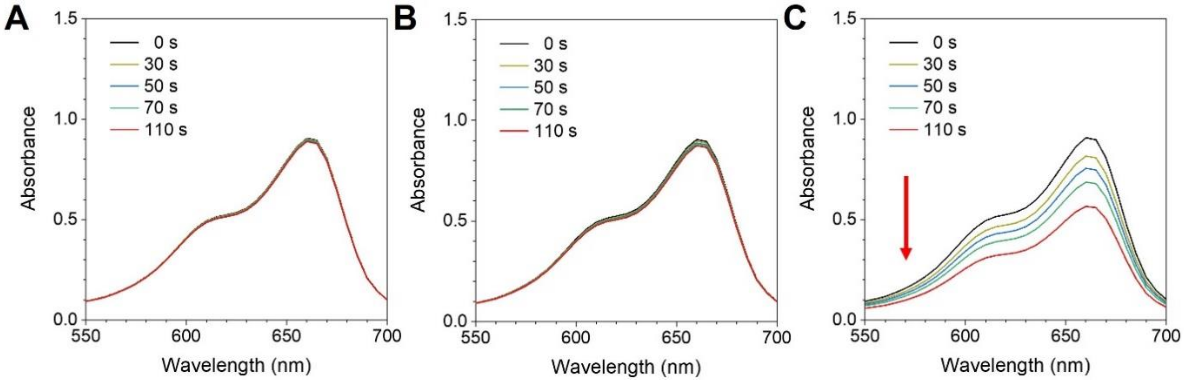

Figure S6. Absorption spectrum of methylene blue in the (A) PBS under laser irradiation and TDCAc solution in the (B) absence or (C) presence of irradiation for different times.

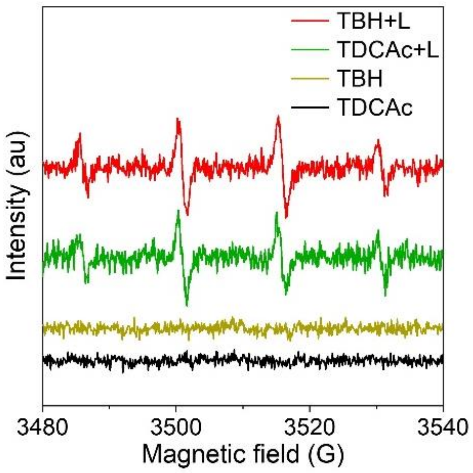

Figure S7. ESR spectrum of TBH and TDCAc in the absence or presence of light irradiation.

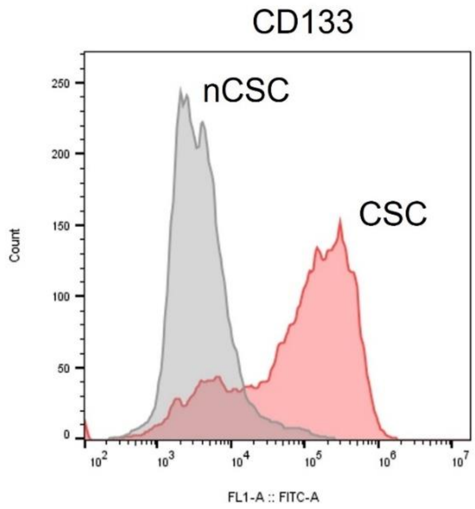

Figure S8. The expression level of CD133 on the surface of nCSC and CSC after cell extraction.

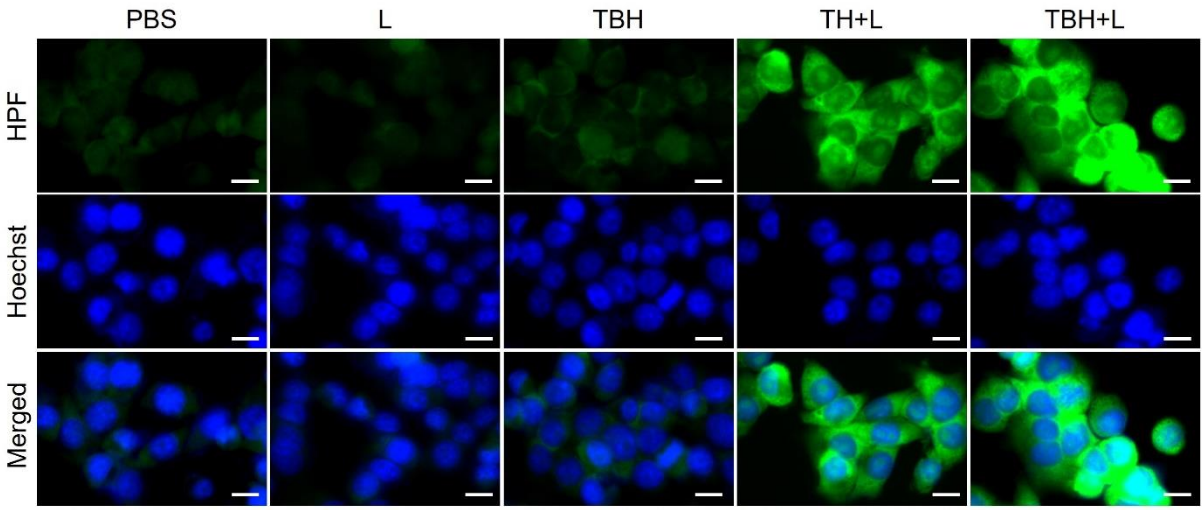

Figure S9. Full confocal imaging of intracellular ROS result in Figure 3C, after treatment with PBS (control) and different formulations with or without 660 nm laser irradiation (0.5W/cm^2, 5 min). Scale bar: 15 μm. Cells were stained with HPF and Hoechst, represented as green and blue in the images.

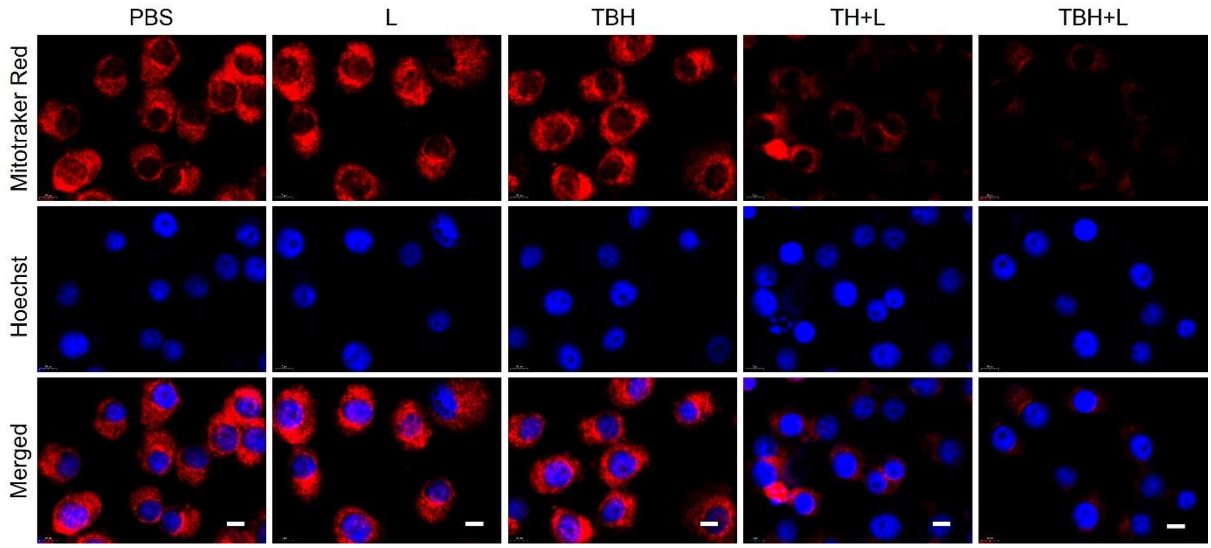

Figure S10. Full confocal imaging of mitochondria staining result in Figure 3D, after treatment with PBS (control) and different formulations with or without 660 nm laser irradiation (0.5W/cm^2, 5 min). Cells were stained with Mitotraker Red and Hoechst, represented as red and blue in the images. Scale bar: 10 μm. Each column shares the same scale bar.

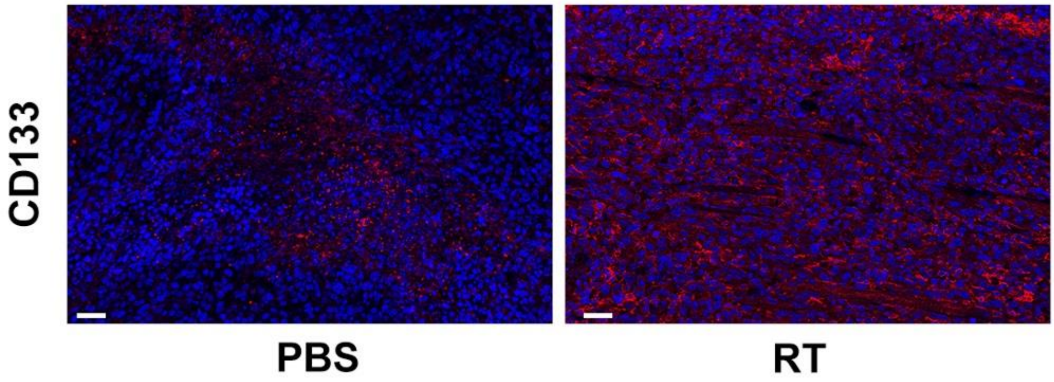

Figure S11. CD133 staining of tumor slices collected in the absence or presence of RT. Scale bar: 40 μm.

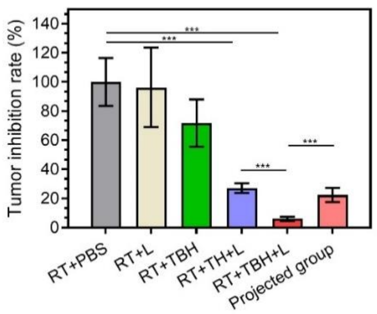

Figure S12. The tumor inhibition rate after various treatments. The tumor inhibition rate = (the tumor weight after treatments)/(the tumor weight after "RT+PBS" treatment) × 100%. The projected additive value is calculated by multiplying the tumor inhibition rate after the RT+TBH treatment and that after RT+TH+L treatment. Data are presented by mean ± SD, n = 5, ***p< 0.001.

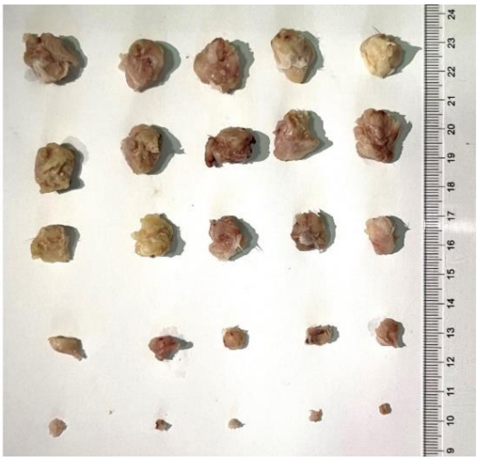

Figure S13. Photographs of the tumors extracted from the mice after the treatment described in Figure 5.

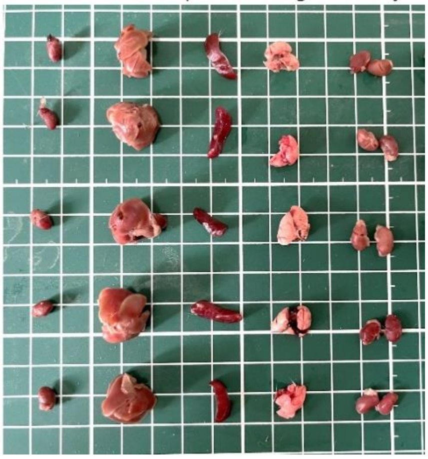

Figure S14. Photographs of the tumors extracted from the mice after the treatment described in Figure 5.

Reference

1. Zhang, T.; Liu, Z.; Tang, W.; Zhu, D.; Lyu, M.; Lam, J. W. Y.; Huang, Q.; Tang, B. Z., Mitochondria-targeting Type I AIE photosensitizer combined with H2S therapy: Uninterrupted hydroxyl radical generation for enhancing tumor therapy. *Nano Today* **2022**, *46*, 101620.

2. Shen, W.; Liu, W.; Yang, H.; Zhang, P.; Xiao, C.; Chen, X., A glutathione-responsive sulfur dioxide polymer prodrug as a nanocarrier for combating drug-resistance in cancer chemotherapy. *Biomaterials* **2018**, *178*, 706-719.

3. Xie, X.; Jiang, K.; Li, B.; Hou, S.; Tang, H.; Shao, B.; Ping, Y.; Zhang, Q., A small-molecule self-assembled nanodrug for combination therapy of photothermal-differentiation-chemotherapy of breast cancer stem cells. *Biomaterials* **2022**, 121598.
